# Is Narrative Enhancement Possible in Healthy Aging? A Preliminary Investigation into the Effects of Advanced Training Activities

**DOI:** 10.1007/s10936-026-10317-x

**Published:** 2026-10-23

**Authors:** S. D’Ortenzio, I. Gabbatore, F. Petriglia, G. Gasparotto, G. Vegna, A. Marini, F. M. Bosco

**Affiliations:** 1 Cognitive Neuroscience Laboratory, University of Udine, Udine, Italy; 2 Department of Humanities, University of Turin, Turin, Italy; 3 GIPSI—research Group on Inferential Processes in Social Interaction, University of Turin, Turin, Italy; 4 Department of Psychology, University of Turin, Turin, Italy; 5 Neuroscience Institute of Turin, University of Turin, Turin, Italy; 6 Scientific Institute IRCCS E. Medea, Pasian di Prato, Udine, Italy

**Keywords:** Training activities, Healthy aging, Discourse production, Narrative enhancement

## Abstract

Over the past two decades, age-related declines have been documented in both the micro- and macrolinguistic aspects of language processing, adversely affecting discourse production in older adults. The present study investigated the potential for enhancing narrative abilities in healthy aging through the Advanced Cognitive Pragmatic Treatment (Advanced-CPT), an intervention designed to improve narrative and communicative abilities by targeting multiple aspects of social interaction. Nineteen healthy Italian-speaking adults aged 65–86 years participated in the program, which consisted of nine sessions lasting approximately 90 min each. Narrative assessments were conducted before training (T0), immediately after training (T1), and at a three-month follow-up (T2) using standardized tasks based on two single‑scene pictures and two cartoon‑sequence stories, administered individually to each participant. Oral productions were transcribed and analyzed using multilevel discourse analysis. The results revealed significant improvements in both microlinguistic and macrolinguistic skills, particularly through reductions in morphological and local coherence errors. Participants also produced more complete sentences and demonstrated greater lexical informativeness following training. Although total word production decreased at follow-up, this pattern was interpreted as reflecting more efficient communication rather than reduced productivity. Overall, the findings suggest that targeted intervention can enhance narrative abilities in healthy aging and support the potential of structured narrative training to promote communicative competence. These results provide new avenues for enhancing quality of life through structured narrative training interventions.

## Introduction

Over the past 20 years there has been growing interest in understanding how healthy aging affects language production (e.g., Marini, 2022; Wright, 2016). Regarding narrative discourse production, age-related declines have been observed in both micro- and macrolinguistic aspects of language processing (Hilviu et al., 2025; Marini et al., 2025; Pistono et al., 2017; Rabaglia & Salthouse, 2011; Sherratt & Bryan, 2019; Thornton & Light, 2006). Microlinguistic processes encompass the lexical and grammatical components of language, whereas macrolinguistic processes involve pragmatic ability and the integration of meaning across utterances. In a recent study, Marini et al. (2025) investigated discourse production in 256 Italian-speaking healthy adults aged 20–89 years, divided into four age groups: young (20–39 years), middle-aged (40–59 years), mature (60–74 years), and older adults (75–89 years). The findings showed an age-related decline across several aspects of discourse production. Speech rate decreased linearly with age, with young adults producing narratives more rapidly than the other groups. At the microlinguistic level, older adults produced significantly more phonological and semantic errors and fewer complete sentences than younger participants. At the macrolinguistic level, the older group exhibited more cohesion and local coherence errors. Global coherence showed a non-linear pattern, declining from young to middle-aged adults, remaining stable between the middle-aged and mature groups, and then decreasing sharply in the oldest group. Similarly, lexical informativeness declined significantly only in the oldest group.

Discourse production plays a crucial role in supporting autonomy of older individuals. The available evidence on age-related language decline highlights the need not only for accurate assessment procedures (see e.g., Arcara & Bambini, 2016; Bischetti et al., 2024, 2025; Hilviu et al., 2022, 2025; Marini et al., 2026) but also for interventions specifically designed to enhance these abilities in the elderly. Regarding this, a useful starting point is provided by rehabilitation programs developed for persons with language impairments, such as aphasia (Davis & Wilcox, 1985; Helm-Estabrooks & Ramsberger, 1986; Thompson & Shapiro, 2005; see Dipper et al., 2021 for a review). Some of these approaches adopt a linguistic perspective and target language processes, primarily grammatical abilities, such as the Treatment of Underlying Forms (TUF; Thompson & Shapiro, 2005) and the Helm Elicited Language Programme for Syntax Stimulation (HELPSS; Helm-Estabrooks & Ramsberger, 1986). Others adopt a functional perspective, emphasizing the ability to convey informative messages, such as the Promoting Aphasics’ Communicative Effectiveness treatment (PACE; Davis & Wilcox, 1985). Although these interventions have demonstrated beneficial effects in people with aphasia (e.g., Marini et al., 2007; Murray et al., 2007), they have generally focused on speech productivity and lexical and grammatical abilities, with comparatively little attention paid to discourse-level informativeness.

The efficacy of these interventions in improving macrolinguistic skills remains largely unexplored, as only a few studies have specifically targeted discourse-level abilities in the elderly. For example, Bambini et al. (2020) implemented an intervention to improve pragmatic skills in Italian-speaking healthy adults aged 60–83. Thirty-two participants received pragmatic training (PragmaCom), whereas 17 underwent cognitive training. Cognitive, verbal, and pragmatic measures were assessed before and after the six-week intervention. The PragmaCom program was designed to enhance communicative competence by drawing on Grice’s conversational maxims. Conducted in groups, the training required participants to identify communicative mismatches in stories and discuss the pragmatic principles that had been violated. By contrast, the cognitive training focused on memory, processing speed, and reasoning. Although both groups improved over time, the effects were larger and more consistent in the PragmaCom group, which also showed a reduction in off-topic verbosity. These results suggest that pragmatic training may be more effective than general cognitive training in enhancing communicative abilities in healthy older adults.

Compared with Bambini et al.’s (2020) functional protocol, linguistic approaches often have lower ecological validity, target isolated language components, and employ activities that do not closely resemble everyday communication. Consequently, their benefits may generalize less readily to everyday communicative contexts and may be more variable across individuals, particularly among those with greater communicative or cognitive declines (Murray et al., 2007). This is consistent with Davis and Wilcox’s (1985) observation that the more naturalistic the intervention, the greater the likelihood that treatment gains will generalize beyond the training context.

Considering that healthy aging affects both micro- and macrolinguistic skills (Marini et al., 2026), we hypothesized that a functionally oriented intervention would be particularly well suited to improving discourse production in older adults. Specifically, we expected that a communication-based training program would enhance language production, with the greatest improvements occurring in macrolinguistic abilities and lexical informativeness. Furthermore, given the close interdependence between micro- and macrolinguistic processes reported by Marini et al. (2025) and Sherratt (2007), we also hypothesized that gains in macrolinguistic abilities would be accompanied by improvements in microlinguistic performance.

To summarize, the primary aim of this study was to evaluate the effects of the Advanced Cognitive Pragmatic Treatment (Advanced-CPT; Gabbatore et al., 2026) on productivity and on micro- and macrolinguistic skills in healthy older adults. We hypothesized that the intervention would improve productivity as well as micro- and macrolinguistic abilities. Additionally, based on evidence that clinical populations retain improvements after training (Braden et al., 2010; McDonald et al., 2008; Dahlberg et al., 2007; Gabbatore et al., 2017), we hypothesized that training effects would persist at the three-month follow-up.

## Methods

### Participants

Nineteen healthy Italian-speaking adults aged between 65 and 86 years participated in the study. Participants’ details are presented in Table 1. All participants scored above the clinical cut-off on the Montreal Cognitive Assessment (MoCA; Conti et al., 2015), the short-form of the Token test (De Renzi & Vignolo, 1962), and the Naming subtest of the Aachener Aphasie Test (AAT; Huber et al., 1984). Exclusion criteria included a current or past diagnosis of psychiatric or neurological disorders, severe visual or hearing impairments, a history of prolonged substance abuse, and the use of mood stabilizers.

**Table 1:** Additional participant information at T0 including results at the montreal cognitive assessment, the short version of the token test, and the naming subtest of the aachener aphasie test

Demographics	M (SD)
Age (years)	71.90 (6.15)
Education (years)	13.79 (2.64)
	*Frequency*
Sex	F = 10 (53%)
Cut-off tasks scores	*M (SD)*
MoCA	24.89 (2.26)
Token test	4.92 (0.19)
AAT	116.53 (3.69)

Participants were recruited in collaboration with cultural and social organizations in Università delle Tre Età, Nichelino and Associazione Solidarietà Volontariato a Domicilio Onlus, as well as through flyers and public engagement activities conducted in the local area. Participants were assessed individually at three time points: before the intervention (T0), immediately after its completion (T1), and at a three-month follow-up (T2). The study was approved by the Bio-Ethical Committee of the University of Turin (protocol n. 0193556) and all the activities were conducted in accordance with the Helsinki Declaration. Before the study, all participants were informed about the aims of the study and provided their written consent. They were also told that their participation was voluntary, and withdrawal was allowed at any time.

### Narrative Production Task

The interaction between micro- and macrolinguistic processes in language production was assessed using the MultiLevel discourse Analysis procedure (MLA; Marini et al., 2011; see Marini et al., 2026 for its recent standardization), which provides a comprehensive assessment of both micro- and macrolinguistic skills. The procedure has been shown to effectively capture language productivity as well as lexical, grammatical, and macrolinguistic abilities in healthy aging (D’Ortenzio et al. 2025; Marini et al. 2005, 2025; Petriglia, Gasparotto et al., 2025; Petriglia, Marini et al., 2025) and is increasingly being adopted in both clinical and research settings (Andreetta et al., 2025; Bryant et al., 2016; Gobbo et al., 2026).

To elicit discourse samples of approximately 300–400 words (a length considered sufficient to ensure adequate test-retest reliability; Nicholas & Brookshire, 1993), four picture stimuli were administered. These included two single-scene pictures (the “Picnic” by Kertesz, 1982; and the “Cookie Theft” picture by Goodglass & Kaplan, 1972) and two cartoon-picture sequences (the “Flowerpot” by Huber & Gleber, 1982; and the “Nest Story” by Paradis & Libben, 1987). The two types of stimuli were selected to elicit different discourse genres. The “Picnic” picture primarily elicits descriptive discourse, as it presents all relevant information simultaneously. In contrast, the “Cookie Theft” picture is more likely to elicit narrative discourse, requiring participants to infer preceding and subsequent events to construct a coherent story. Cartoon-picture sequences, on the other hand, are widely used by clinicians to elicit narrative discourse, as they provide an underlying story structure that participants must integrate using coherent language and sentence organization.

All picture stimuli and cartoon stories were presented in a randomized order on a laptop screen positioned in front of the participant, thereby preventing referent sharing with the examiner. To minimize the impact of potential short-term memory demands, each stimulus remained visible throughout the narrative production task. Before the assessment began, participants were informed that the experimenter was unfamiliar with the stimuli and were instructed to describe each image as clearly as possible while avoiding ambiguous referential expressions. A message was displayed on the screen and read aloud by the experimenter: “Now you will see stories in the form of images. The images are randomly selected by the program, so I don’t know them. As soon as the image appears, you will need to describe it to me. There is no time limit and no right or wrong way to describe them. You can speak as much or as little as you like. However, I ask that you avoid using words like ‘here,’ ‘this,’ etc., and try to be as clear as possible. When you’re finished, press the space bar, and the program will select the next story.” All narratives were audio-recorded for subsequent transcription and analysis.

### Assessment of Narrative Discourse Production

Speech samples were transcribed using a semi-automatic procedure developed by one of the authors (F.P.). To facilitate transcription procedures and subsequent analyses, an automated Python workflow was implemented in Google Colab. The initial transcripts were generated using the Whisper large-v3 model (Radford et al., 2022) and further processed using the SpaCy Python library (Honnibal et al., 2020) for lemmatization and part-of-speech tagging. As Whisper tends to omit repetitions, self-repairs, and false starts, a human reviewer edited the transcripts to manually integrate these missing elements. The reviewer also resolved ambiguities in word classification. The final transcripts included the total duration (in seconds) of each narrative, phonological fillers, pauses, false starts, phonological errors, and neologisms. Utterances were segmented based on acoustic, semantic, grammatical, and phonological criteria, following the guidelines described in Marini et al. (2011).

In accordance with the multilevel discourse analysis framework proposed by Marini et al. (2011), the transcripts were analyzed across three domains (productivity, microlinguistic, and macrolinguistic efficiency), yielding ten narrative measures.

*Productivity* was assessed by measuring the total number of phonologically well-formed words (excluding false starts, phonological paraphasias, and neologisms), and speech rate, calculated as words per minute using the formula: ([number of words/duration in seconds]*60).

*Microlinguistic* measures included percentages of phonological, morphological, and semantic errors and a percentage of complete sentences. Phonological errors included all speech units that are not considered as phonologically well-formed words, such as false starts, phonological paraphasias, and neologisms. False starts are interrupted attempts at word production. Phonological paraphasias are words with phonemic distortions due to inversion, omission, insertion, or substitution of phonemes (e.g., *methar, *mothe, *nother instead of *mother*). Neologisms are unrecognizable words (e.g., “glot” instead of *dog*). The percentage of phonological errors was calculated by dividing the number of such errors by the total number of speech units multiplied by 100. Morphological errors involved incorrect use of morphemes or morphosyntactic structures such as subject-verb agreement errors or misuse of function words (e.g., articles, prepositions, conjunctions). The percentage of morphological errors was calculated by dividing the number of such errors by the total number of well-formed words multiplied by 100. Semantic errors were defined as the substitution of target words with semantically related but incorrect alternatives (e.g., saying *newspaper* instead of *book*). The percentage of semantic errors was calculated by dividing the number of such errors by the total number of well-formed content words multiplied by 100. All well-formed sentences, excluding those with omissions or morphological errors were considered as correct sentences. The percentage of complete sentences was calculated by dividing the number of utterances that were scored as complete sentences by the total number of utterances multiplied by 100.

*Macrolinguistic* measures included the percentage of local coherence errors, two measures of global coherence errors (i.e., percentages of tangential utterances and percentages of erroneous semantic formulations), and the percentage of lexical informativeness. *Local coherence* refers to the conceptual connection between successive utterances. Errors occurred when a referent was missing or an utterance was interrupted prematurely without completion, and the next utterance introduced unrelated information (e.g., “*he’s trying to*…/*The girl waits for a cookie*”). The *percentage of local coherence errors* was calculated by dividing the number of these errors by the number of utterances multiplied by 100. *Global coherence* concerns the alignment of utterances semantically with the overall meaning of the narrative. Errors were classified as tangential utterances, which diverge from the main narrative (e.g., “*Two people are having a picnic by the lake*/*I go every Sunday to the beach for a picnic*”), or erroneous semantic formulations, which introduce information that is incongruent with the depicted content (e.g., “*The woman is washing the dishes*/*The kids are helping her*”, when the image actually shows children stealing cookies). Each type of global coherence error was calculated as the number of errors divided by the total number of utterances multiplied by 100. Finally, the analysis of macrolinguistic skills included the percentage of lexical informativeness that was measured as the proportion of words that were not only phonologically well-formed but also appropriate from a grammatical and pragmatic point of view. Words were excluded from this count if they were phonological, semantic, or morphological errors, repetitions or fillers, lacked clear referents, or occurred within tangential or semantically unrelated utterances. The percentage of lexical informativeness was calculated as the number of informative words divided by the total number of words multiplied by 100.

### Advanced Training Activities

The Advanced Cognitive Pragmatic Treatment (Advanced-CPT, Gabbatore et al., 2026) is an intervention program grounded on Cognitive Pragmatics (Bara, 2010), a theory centered on inferential processes, and the appropriate use of language in context. The program directly and indirectly stimulates all the narrative domains examined in the present investigation: productivity, microlinguistic, and macrolinguistic efficiency.

The Advanced-CPT aims to enhance narrative and communicative abilities by targeting various aspects of social interaction. The training specifically focuses on the inferential processes required to understand complex communicative phenomena such as indirect speech acts, irony and deceit. These forms of communication require different levels of inferential abstraction, and the program is designed to strengthen this capacity, enabling individuals to move beyond literal interpretations and better grasp implicit meanings, nuances, and social cues. Several sessions of the program are dedicated to developing narrative skills through speech elicitation and storytelling tasks. In addition, the training emphasizes increasing participants’ self-awareness of their communicative challenges, which is essential for achieving meaningful and lasting improvements. By reflecting on their own communication processes, participants are encouraged to develop a more conscious and adaptive approach to social exchanges.

The Advanced-CPT includes progressively complex activities designed to engage healthy older adults. Comprehension activities include a selection of scenes from movies or short films, providing rich and realistic communicative scenarios. Production activities primarily include role-playing tasks that simulate everyday situations and are structured to engage multiple participants simultaneously, fostering social interaction and collaboration. Similarly, activities focused on prosodic cues adopt a multimodal approach, integrating vocal tone, facial expressions, and body language to enhance expressiveness and communication effectiveness.

A key innovation of the Advanced-CPT is its emphasis on intergenerational communication, specifically targeting participants’ ability to interpret idiomatic expressions, metaphors, and colloquialisms commonly found in contemporary language. Because misunderstandings in these areas often hinder effective interaction, this aspect of the program is particularly valuable for promoting effective and fluid communication. The program also addresses the challenges of digital communication (e.g., email, instant messaging, and social media) helping participants use these tools more appropriately and effectively.

The training consists of nine weekly sessions, each lasting approximately 90 min, with a 10-minute break. Sessions are conducted in small groups of 5–6 participants and are led by master’s students in psychology, under the supervision of experienced psychologists and psychotherapists.

Throughout the sessions, self-monitoring and feedback from both facilitators and peers play a central role in reinforcing progress and deepening participants’ understanding of their communicative behavior. All sessions are video-recorded (with participants’ consent), providing a valuable resource for reflection and analysis. Video feedback, offered both during and after the program, is a key element in fostering greater self-awareness and helping participants monitor their progress over time.

### Statistical Analyses

*Power analysis.* Sample size was determined a priori using G*Power (Faul et al., 2007), based on the results reported in Gabbatore et al. (2026). In that study, the training was administered to a sample of healthy older adults, and pragmatic ability was assessed across three time points. All observed effects were equal to or greater than a partial eta squared of approximately 0.10. The present study replicates the same experimental design, replacing pragmatic assessment with the evaluation of narrative discourse. Pragmatic competence and discourse production are distinct but partially overlapping dimensions of communicative functioning. (Petriglia, Marini et al., 2025). In the absence of preliminary estimates of the training effect on narrative outcomes, the effect size observed for pragmatic ability in the previous study was used as the most closely related available reference for sample size estimation. Assuming η_p_² = 0.10 (corresponding to Cohen’s f = 0.33), an a priori power analysis indicated that a total sample of 16 participants would be required to achieve 80% power, whereas 18 participants would be needed to achieve 85% power.

To examine the effects of the intervention on the ten narrative measures, a series of one-way repeated-measures ANOVAs were conducted with assessment session (T0, T1, and T2) entered as the within-subjects factor. When the assumption of normality was violated, training effects were assessed using Friedman’s test. When the assumption of sphericity, assessed using Mauchly’s test, was violated, the degrees of freedom for the F-test were adjusted using the Greenhouse-Geisser or the Huynh-Feldt epsilon (ε) correction, as appropriate. Post hoc pairwise comparisons were performed using the Holm-Bonferroni method for parametric analyses and Conover’s post hoc test following Friedman’s tests. All statistical analyses were conducted using JASP (v. 0.98; JASP Team, 2026).

## Results

The results of the speech samples collected at T0 (before training), T1 (after training), and T2 (follow-up) are presented in Table 2.

**Table 2:** Means and standard deviations for the narrative measures at baseline (T0), post-intervention (T1), and follow-up (T2)

	T0 M (SD)	T1 M (SD)	T2 M (SD)	Post hoc comparison
Words*	724.68 (370.90)	829.05 (413.60)	648.90 (375.73)	T1 > T2
Speech rate	127.17 (20.56)	133.76 (22.93)	131.09 (21.69)	
% Phonological errors	1.36 (0.62)	1.30 (0.71)	1.10 (1.00)	
% Semantic errors	1.46 (1.47)	0.58 (0.49)	0.93 (0.86)	
% Morphological errors*	2.73 (2.86)	1.84 (1.52)	1.16 (1.40)	T0 > T2
% Complete sentences*	65.44 (9.40)	71.46 (8.99)	70.97 (8.78)	T0 < T1
% Local coherence errors*	21.79 (9.32)	14.38 (6.76)	19.44 (9.43)	T0 > T1< T2
% Tangential utterances*	1.38 (3.00)	0.30 (1.23)	0.33 (1.44)	T0 > T1=T2
% Erroneous semantic formulations*	1.62 (2.26)	0.10 (0.29)	0.10 (0.30)	T0 > T1=T2
% Lexical informativeness*	77.18 (8.08)	80.92 (8.78)	81.28 (6.37)	

Words refers to the mean number of words produced by each participant across the discourse elicitation tasks

The asterisk (*) shows when a statistically significant condition-related difference was detected

**Fig. 1:**
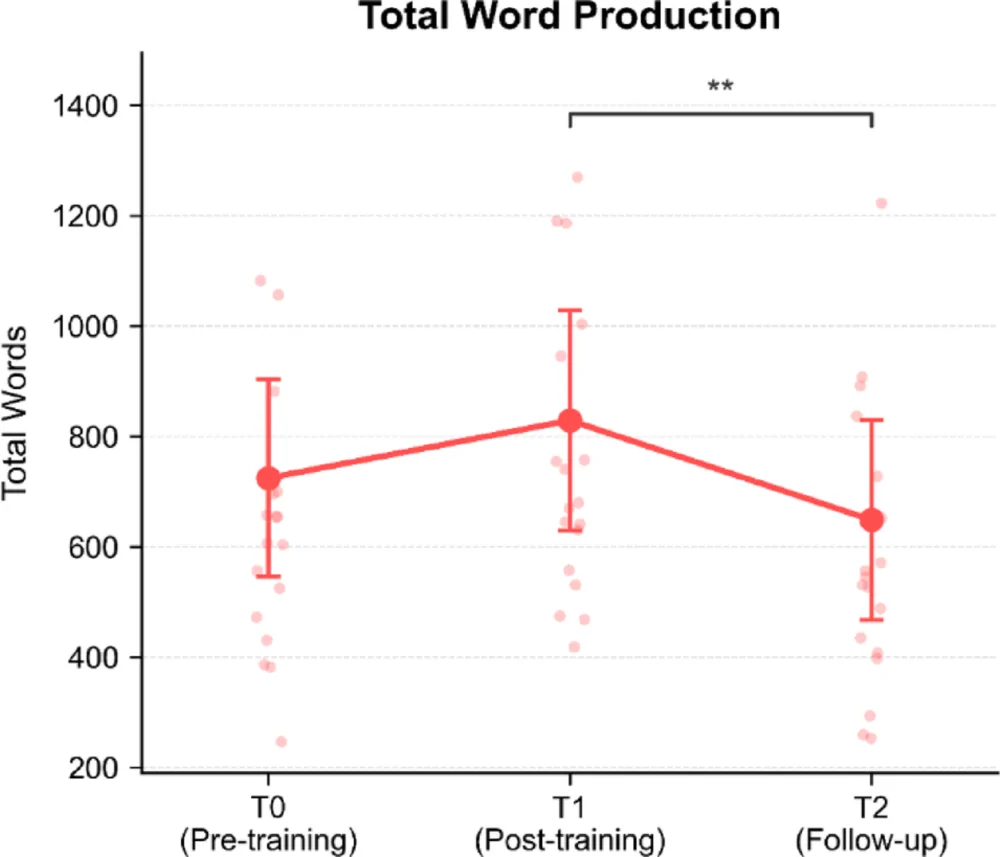
Productivity: words produced across the three sessions

Considering productivity, the repeated measures ANOVA showed that the effect of condition on *word production* was significant (F[2, 36] = 4.162, *p* =.024, η_p_² = 0.188) with a quadratic trend (*p* = .009). Holm-Bonferroni post-hoc analyses showed that at T1 participants produced significantly more words than at T2 (*p* = .005). No differences were found for the other contrasts (see Fig. 1). No significant difference was found between the three conditions in speech rate (F[2, 36] = 1.078, *p* =.351, η_p_² = 0.056).

**Fig. 2:**
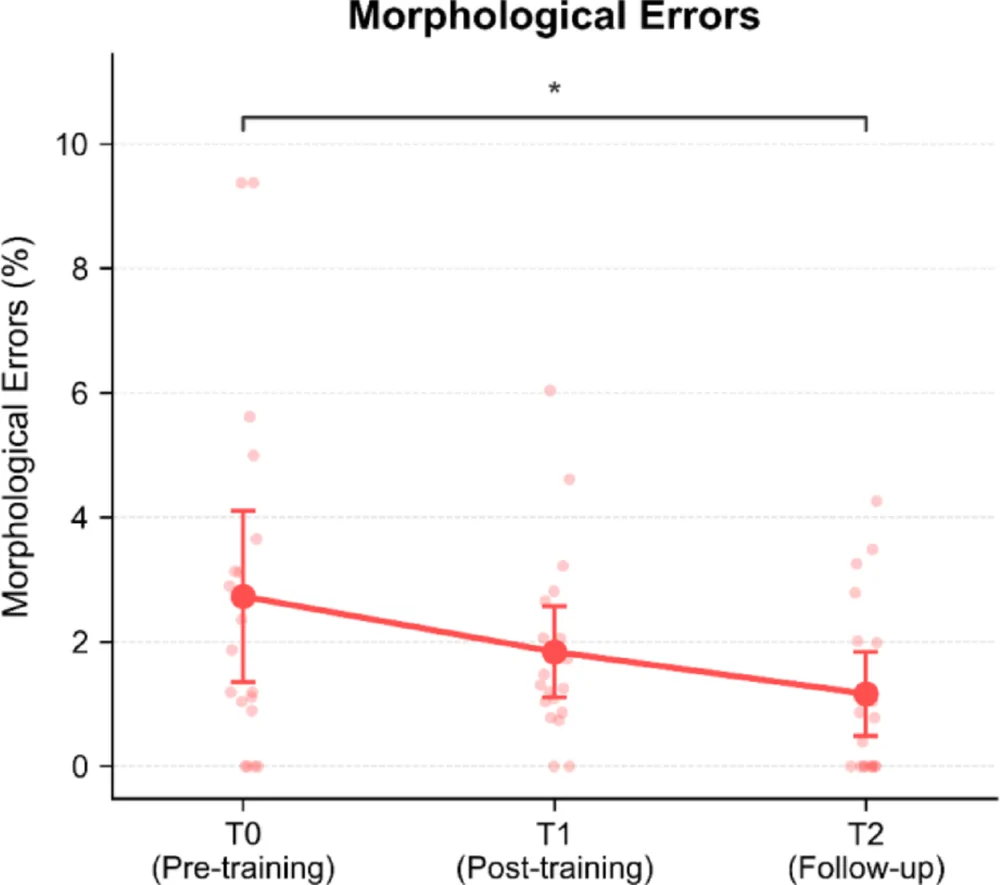
Microlinguistic analysis: percentage of morphological errors produced across the three sessions

**Fig. 3:**
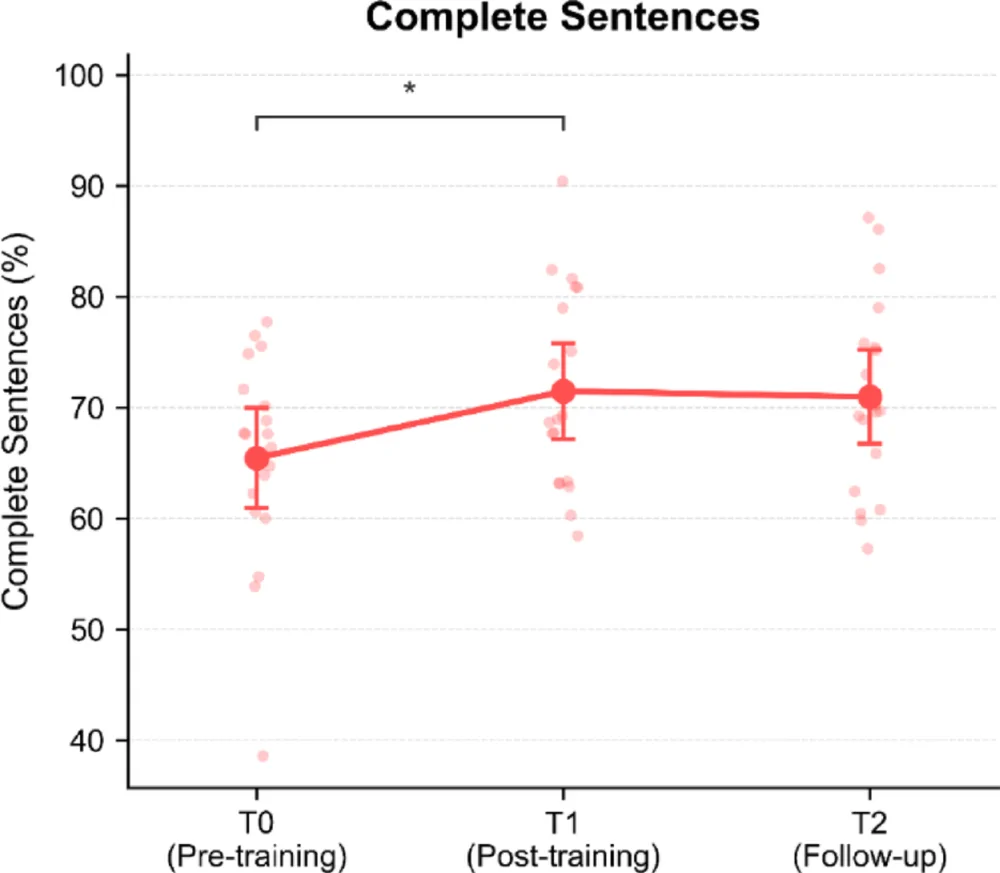
Microlinguistic analysis: percentage of complete sentences produced across the three sessions

Considering microlinguistic measures, no session-related difference was found in the production of % Phonological Errors (X^2^_F_[2] = 1.684, *p* =.431, Kendall’s W = 0.044). Similarly, the Friedman test did not show a significant effect of condition on % Semantic errors (X^2^_F_[2] = 5.108, *p* =.078, Kendall’s W = 0.134). However, the Friedman test showed that the effect of condition on % Morphological errors was significant (X^2^_F_[2] = 6.113, *p* =.047, Kendall’s W = 0.161) with a linear trend (*p* =.048). Conover’s post hoc comparison showed that at T2 participants produced fewer morphological errors than at T0 (*p* =.042). No differences were found for the other contrasts (see Fig. 2). As for the % Complete sentences (see Fig. 3), the repeated measures ANOVA showed that the effect of training was significant (F[2, 36] = 4.022, *p* =.027, η_p_² =0.183) with a linear trend that approached significance (*p* =.056). Holm post hoc analyses showed that at T1 participants produced more complete sentences than at T0 (*p* =.044). No differences were found for the other contrasts.

**Fig. 4:**
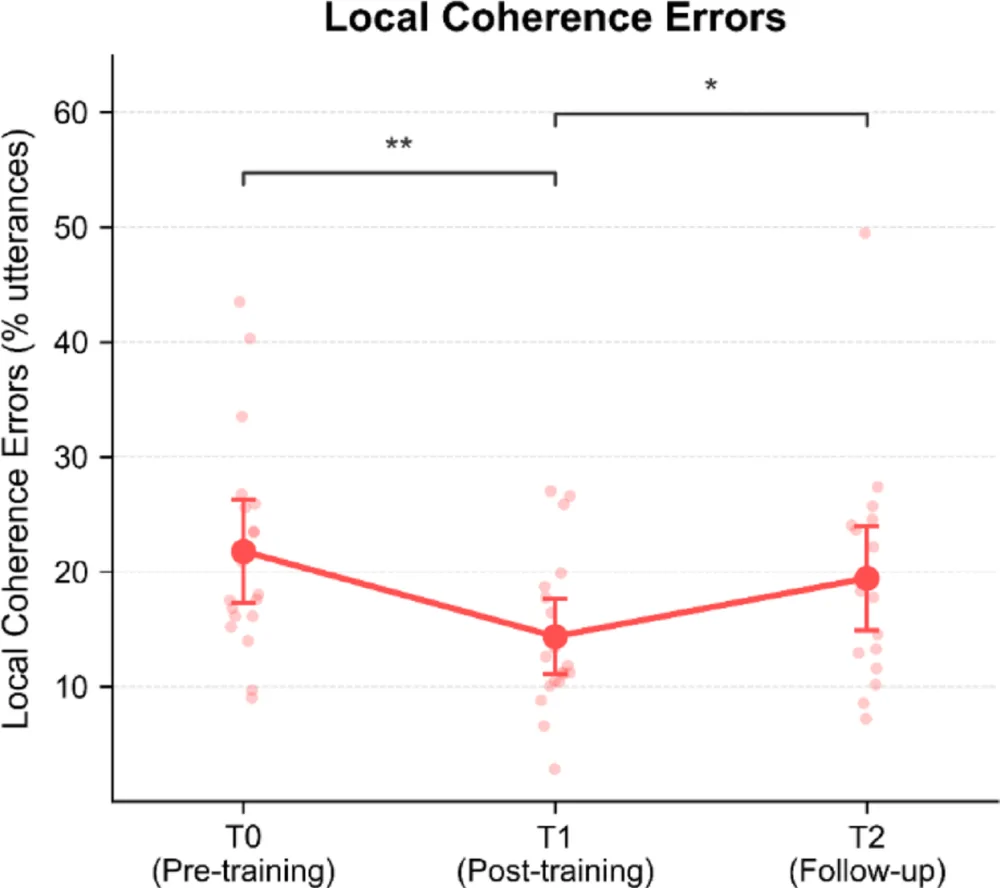
Macrolinguistic analysis: percentage of local coherence errors produced across the three sessions

Regarding the assessment of macrolinguistic skills, the repeated measures ANOVA showed that the effect of condition on % Local coherence errors was significant (F[2, 36] = 7.168, *p* =.002, η_p_² =0.285) with a quadratic trend (*p*<.001) (see Fig. 4). Holm post hoc analyses showed that the participants at T0 produced significantly more % Local coherence errors than at T1 (*p* =.002). Post hoc analyses also showed that participants at T2 produced significantly more % Local coherence errors than at T1 (*p* =.016) but the same number of such errors compared to T0 (*p* =.346).

**Fig. 5:**
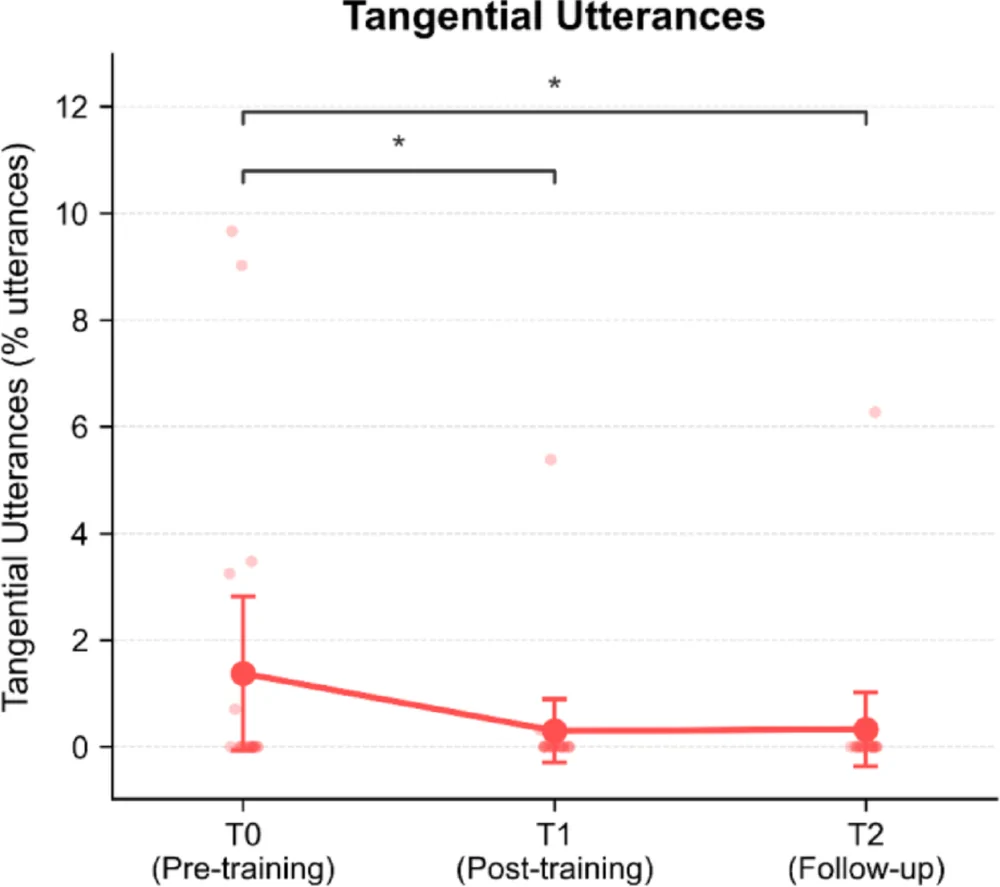
Macrolinguistic analysis: percentage of tangential utterances produced across the three sessions

As for the % Tangential utterances, the Friedman’s test showed a significant effect on the production of tangential sentences (X^2^_F_[2] = 8.824, *p* =.012, Kendall’s W = 0.232) (see Fig. 5). Conover’s post hoc analyses showed that at T0 participants produced more tangential utterances than at T1 (*p* =.007) and at T2 (*p* =.007).

**Fig. 6:**
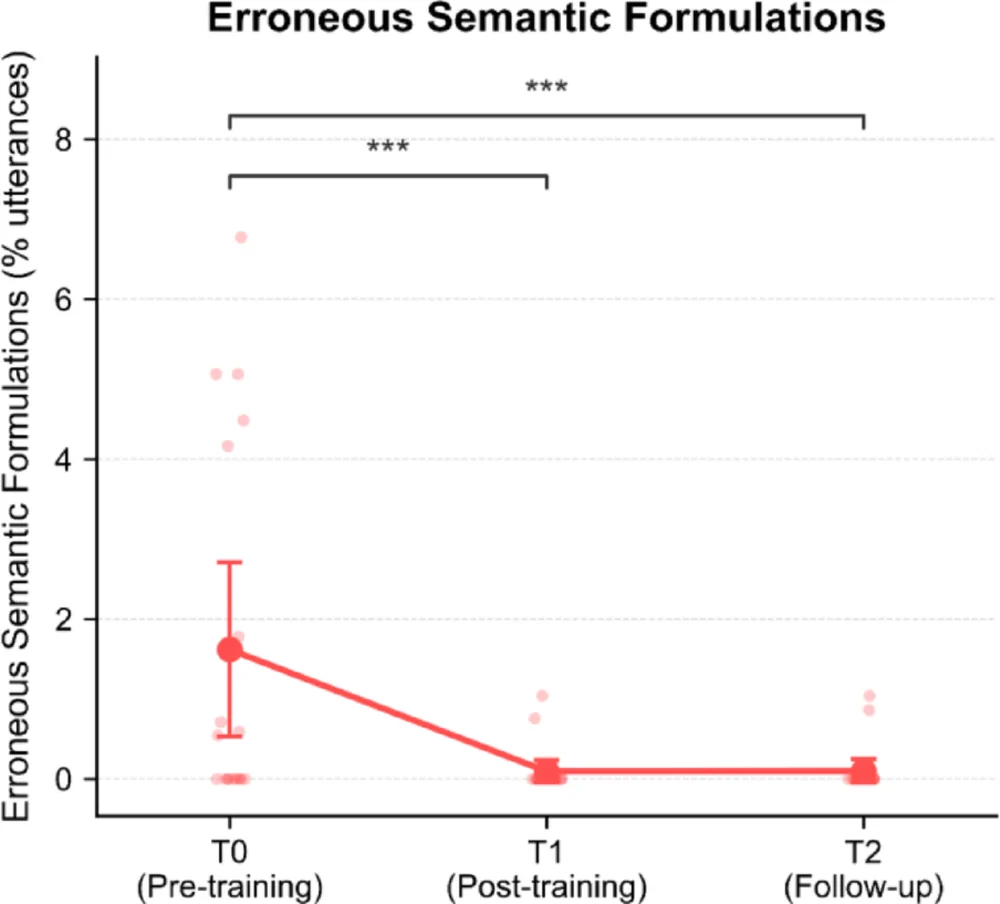
Macrolinguistic analysis: percentage of erroneous semantic formulations produced across the three sessions

Similarly, Friedman’s test showed that the effect on % Erroneous semantic formulations was significant (X^2^_F_[2] = 15.060, *p* <.001, Kendall’s W = 0.396) with both linear (*p* =.009) and quadratic (*p* =.003) trends (see Fig. 6). Conover’s post hoc analyses showed that at T0 participants produced more erroneous semantic formulations than at T1 (*p* <.001) and at T2 (*p* <.001).

**Fig. 7:**
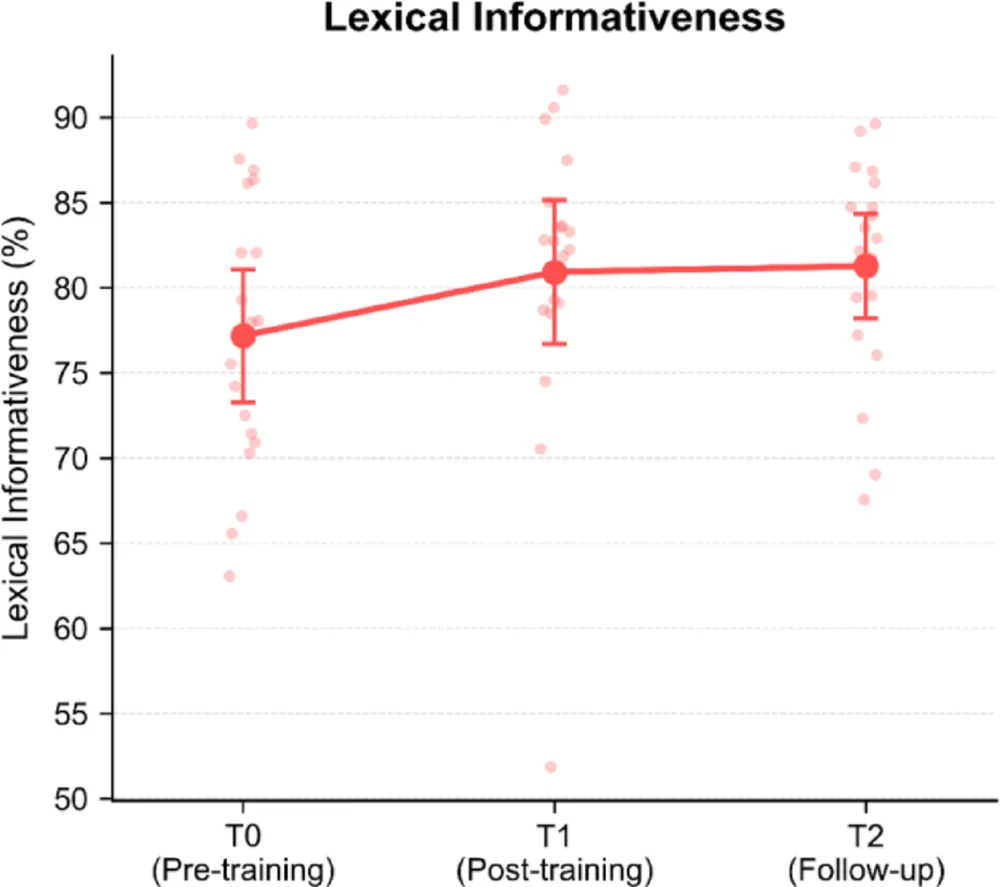
Macrolinguistic analysis: percentage of lexical informativeness across the three sessions

For lexical informativeness, the repeated-measures ANOVA revealed a significant effect of condition, F(2, 36) = 3.432, *p* =.043, η_p_² = 0.160. Trend analysis further indicated a significant linear increase across the three assessment points (*p* =.029) (Fig. 7), suggesting a progressive improvement over time. Although none of the pairwise post hoc comparisons reached significance after correction for multiple comparisons, the significant linear trend indicates that lexical informativeness increased systematically across the study period.

## Discussion

In this study we analyzed the efficacy of an advanced training program designed to enhance communicative skills in healthy older adults, focusing on its potential effects on discourse production and, especially, macrolinguistic abilities. The theoretical rationale for the intervention was grounded in the close interdependence between macro- and microlinguistic processes in discourse production (Marini et al., 2025; Sherratt, 2007). Accordingly, we hypothesized that the intervention would enhance macrolinguistic skills with downstream benefits for microlinguistic processing and language productivity, and that such enhancement would be maintained at the three-month follow-up. Our hypotheses were confirmed by the results.

From a linguistic perspective, the training positively affected productivity as well as both micro- and macrolinguistic performance. Regarding productivity, the training did not increase speech rate. However, a significant reduction in the total number of words produced during storytelling was detected at follow-up relative to post-training. As this reduction was accompanied by fewer morphological errors and improved macrolinguistic skills, it likely reflects a shift toward more efficient and effective communication rather than a decline in productivity. In other words, participants appeared to become less verbose while communicating more efficiently. At the microlinguistic level, the training did not influence the production of phonological and semantic errors but significantly improved the application of morphological rules during word inflection. This improvement was likely associated with the increased production of complete sentences observed across the three sessions. Consistent with the findings of Thompson et al. (1997) in people with aphasia, participants produced fewer morphological errors at follow-up than at baseline. Furthermore, grammatical production had already improved immediately after the intervention and remained stable at follow-up, suggesting a lasting positive effect on sentence construction abilities. The analysis of macrolinguistic aspects of discourse production also revealed beneficial effects of the intervention. Following training, participants produced significantly fewer local coherence errors, indicating an improved ability to select words with clear referents while taking the interlocutors’ informational needs into account. This suggests greater attention to referential clarity during discourse production. However, local coherence errors increased again at follow-up, resulting in more errors than at T1 and no significant difference from baseline. This pattern suggests that this improvement was not fully maintained over time and may have been more closely related to the immediate effects of the training.

For lexical informativeness, the repeated-measures analysis showed a significant effect of condition with a significant linear trend; no pairwise comparison survived correction. Since the three conditions form an ordered sequence (pre-training, post-training, follow-up), the linear contrast is the appropriate test here. Its significance indicates a steady increase in lexical informativeness, consistent with a training-related improvement in the informativeness of participants’ output. However, the effect was modest and the associated p-values were close to the conventional threshold; therefore, the result should be interpreted with caution. The study also lacked an untrained control group. Therefore, the observed improvement cannot be attributed solely to the intervention. Alternative explanations, including practice or test–retest effects arising from repeated administration and increasing familiarity and engagement with the trainer across sessions, cannot be excluded. Future work with a control condition and a larger sample would help to disentangle genuine training-induced gains from these more general exposure effects.

Considering global coherence errors, the relatively low rate of tangential utterances and erroneous semantic formulations at baseline decreased significantly following the intervention and remained stably low at follow-up. This provides further evidence that the proposed training can strengthen macrolinguistic abilities in the elderly. Additional support for this interpretation comes from the observed increase in the production of informative words following training.

The results of the present investigation are consistent with those reported by Gabbatore et al. (2026), who observed significant improvements in both pragmatic comprehension and production following the Advanced-CPT. In particular, the improvements in linguistic abilities reported in their study are consistent with the enhancements in discourse production observed here. Improvements across most of the linguistic measures remained stable over time, further supporting the durability of the intervention’s effects.

Moreover, these findings complement previous evidence of broad age-related changes in pragmatic comprehension (see Hilviu et al., 2022; Gabbatore et al. 2025; Vegna et al. 2026), with particularly marked declines in humor (Bischetti et al., 2023) and metaphor comprehension (Ceccato et al., 2025). The present results extend this picture by showing that, against the background of age-related decline in narrative abilities (see Hilviu et al. 2025; Petriglia, Gasparotto et al., 2025; Petriglia, Marini et al., 2025), an advanced communication-oriented intervention may also improve narrative production, which is closely related to pragmatics but involves distinct linguistic and discourse-level mechanisms.

Qualitative observations collected during the sessions also provided some indications regarding participants’ engagement with the different training activities. Role-playing and video-based tasks appeared particularly effective in promoting participation, spontaneity, and group interaction, including among participants who were initially more reserved. The opening discussion, centered on participants’ experiences since the previous session, also appeared to foster reflection on everyday communication and the transfer of training content beyond the sessions. More demanding activities, such as those involving mental flexibility, information selection, and narrative organization, occasionally elicited uncertainty or frustration, but also appeared to foster greater awareness of individual communicative habits. In particular, tasks focusing on clarity, referential precision, and relevance highlighted marked individual differences in verbosity and discourse appropriateness. Finally, some exercises may have been perceived as less challenging by certain participants, particularly those with higher educational attainment or greater prior familiarity with language- and communication-related activities, although they still seemed useful in drawing attention to difficulties that were subsequently recognized in everyday communication. These observations should be considered preliminary, as acceptability and perceived difficulty were not assessed systematically.

Taken together, the findings suggest that the intervention was particularly successful in enhancing higher-level discourse organization and grammatical sentence construction. The most robust gains concerned global coherence, morphological accuracy, sentence construction, and communicative efficiency, whereas speech rate, phonological and semantic errors, and the maintenance of local coherence appeared less responsive. The qualitative observations complement this pattern by suggesting that engagement was greatest in interactive and ecologically meaningful activities, while more demanding tasks may have contributed primarily to increasing awareness of participants’ difficulties and communicative habits.

Despite these encouraging findings, the study has some limitations. First, the absence of a control group prevents a definitive attribution of the observed improvements in narrative abilities to the intervention itself. Some of the gains may instead reflect nonspecific factors, such as repeated testing. Future studies should therefore include an appropriate control condition to disentangle these potential influences. Second, cognitive abilities were not reassessed following the intervention. Evaluating Executive Functions (EFs; Diamond, 2013) (i.e., planning, updating, inhibitory control, and shifting) as well as Theory of Mind (ToM; Premack & Woodruff, 1978), the ability to infer the mental states underlying others’ behavior, would provide a more comprehensive characterization of participants’ cognitive profiles (see also (Merlini et al. 2026). Given the close relationship between these abilities and communication (e.g., Bosco and Gabbatore 2017; Bosco et al. 2018a, b; Parola et al. 2026), evaluating EFs and ToM before and after training would also help determine whether the observed improvements in narrative production are accompanied by broader cognitive changes or are specific to communicative abilities.

Future studies should test the efficacy of the training in clinical aging populations characterized by communicative and narrative difficulties, including individuals with mild cognitive impairment, dementia, and primary progressive aphasia. It would also be interesting to explore whether combining the training with non-invasive brain stimulation techniques may enhance or prolong its effects on narrative and communicative abilities. Considering the effect of socioeconomic status on narrative and procedural discourse in aging (see Sherratt, 2024), future research should also investigate whether socioeconomic status plays a role in facilitating the increase in narrative ability.

Overall, the results of the present investigation are promising and fill a research gap in the literature. The relevance of enhancing narrative ability in healthy aging has been quite underrated, thereby neglecting the potential beneficial effects of such improvement on the participants’ quality of life. The results provide preliminary evidence supporting new intervention strategies to mitigate cognitive and communicative decline in aging, suggesting that narrative abilities can be enhanced in healthy older adults, that these gains remain relatively stable over time, and that they may ultimately contribute to improved well-being in later life.

## Data Availability

The datasets generated during and/or analyzed during the current study are not publicly available for privacy or ethical restrictions; however, data will be available from the corresponding author (ilaria.gabbatore@unito.it) on reasonable request.
